# Development of a physiomimetic model of acute respiratory distress syndrome by using ECM hydrogels and organ-on-a-chip devices

**DOI:** 10.3389/fphar.2022.945134

**Published:** 2022-09-02

**Authors:** Esther Marhuenda, Alvaro Villarino, Maria Narciso, Linda Elowsson, Isaac Almendros, Gunilla Westergren-Thorsson, Ramon Farré, Núria Gavara, Jorge Otero

**Affiliations:** ^1^ Unitat de Biofísica i Bioenginyeria, Facultat de Medicina i Ciències de la Salut, University de Barcelona, Barcelona, Spain; ^2^ CIBER de Enfermedades Respiratorias, Instituto de Salud Carlos III, Madrid, Spain; ^3^ The Institute for Bioengineering of Catalonia, The Barcelona Institute of Science and Technology, Barcelona, Spain; ^4^ Lung Biology, Biomedical Center, Department of Medical Science,Lund University, Lund, Sweden; ^5^ Institut d’Investigacions Biomèdiques August Pi i Sunyer, Barcelona, Spain

**Keywords:** ARDS, lung-on-a-chip, extracellular matrix, hydrogels, mesenchymal stromal cells, alveolar epithelial cells, inflammation

## Abstract

Acute Respiratory Distress Syndrome is one of the more common fatal complications in COVID-19, characterized by a highly aberrant inflammatory response. Pre-clinical models to study the effect of cell therapy and anti-inflammatory treatments have not comprehensively reproduced the disease due to its high complexity. This work presents a novel physiomimetic *in vitro* model for Acute Respiratory Distress Syndrome using lung extracellular matrix-derived hydrogels and organ-on-a-chip devices. Monolayres of primary alveolar epithelial cells were cultured on top of decellullarized lung hydrogels containing primary lung mesenchymal stromal cells. Then, cyclic stretch was applied to mimic breathing, and an inflammatory response was induced by using a bacteriotoxin hit. Having simulated the inflamed breathing lung environment, we assessed the effect of an anti-inflammatory drug (i.e., dexamethasone) by studying the secretion of the most relevant inflammatory cytokines. To better identify key players in our model, the impact of the individual factors (cyclic stretch, decellularized lung hydrogel scaffold, and the presence of mesenchymal stromal cells) was studied separately. Results showed that developed model presented a more reduced inflammatory response than traditional models, which is in line with what is expected from the response commonly observed in patients. Further, from the individual analysis of the different stimuli, it was observed that the use of extracellular matrix hydrogels obtained from decellularized lungs had the most significant impact on the change of the inflammatory response. The developed model then opens the door for further *in vitro* studies with a better-adjusted response to the inflammatory hit and more robust results in the test of different drugs or cell therapy.

## 1 Introduction

Acute Respiratory Distress Syndrome (ARDS), commonly caused by bacterial or viral pneumonia (Matthay et al., 2019), is characterized by lung parenchymal damage from increased endothelial and epithelial permeability (non-cardiogenic pulmonary edema) (Staub 1981). ARDS mortality is approximately 25%–40%, and the only treatment is primarily supportive with lung-protective ventilation (Brower et al., 2000). Major efforts in the medical community are focused mainly on the prevention of the injury (Yadav, Thompson, and Gajic 2017) since, although numerous pharmacologic strategies have been successful in animal studies, few trials have shown a clinical benefit in terms of mortality (Ballard-Croft et al., 2012). On the other hand, cell therapies based on the use of mesenchymal stromal cells (MSCs) have started to show some efficacy. However, the mechanisms involved in the process are still to be determined (Laffey and Matthay 2017). Moreover, during the COVID-19 pandemic, ARDS has been reported as a common complication that dramatically increased the mortality of patients (Wu et al., 2020). The aberrant inflammatory response of these patients (known as the cytokine storm) has been correlated with the severity of the disease and it has become one of the main therapeutic targets (Chen et al., 2020). Nevertheless, neither *in vitro* nor *in vivo* available models realistically recreate the ARDS complex pathophysiology (Huppert and Matthay 2017). Therefore, there is an urgent need to develop models with higher physiological relevance to understand the inflammatory processes related to ARDS, the impact of cell therapy (Nonaka et al., 2020), and the use of anti-inflammatory drugs (Trivedi, Verma, and Kumar 2020). Conventional ARDS *in vitro* models mainly involve applying an inflammatory hit (usually by a bacteriotoxin) to a monolayer of pulmonary epithelial or endothelial cells (Cabrera-Benítez et al., 2016). These conventional models do not fully mimic the complex three-dimensional microarchitecture or the extracellular matrix (Burgess et al., 2016) (ECM) stiffness experienced by cells *in vivo.* Moreover, lung cells *in vivo* are subjected to mechanobiological signals such as those induced by the cyclic stretch associated with breathing or mechanical ventilation.

Two different technologies have recently gained popularity for creating physiomimetic models to recreate the ECM (Busch, Lorenzana, and Ryan 2021) and the mechanobiological signals *in vitro*: 3D cell cultures (Habanjar et al., 2021) and organ-on-a-chip devices (Mertz, Ahmed, and Takayama 2018; Benam, Burgess, and Stewart 2021). Indeed, it has been shown that lung ECM can be obtained by decellularizing the native tissues with detergents and enzymes (Nonaka et al., 2014) and then pulverized and reconstituted in the form of hydrogels suitable for 3D cell culture (Pati and Cho 2017). On the other hand, organ-on-a-chip devices (Bassi et al., 2021) try to recreate the physical microenvironment of living organs *in vitro*. In the specific case of a lung-on-a-chip, devices with control over the cyclic stretch and oxygenation have been developed (Campillo et al., 2016; Huh et al., 2013). In the present work, the aim was to merge these two Frontier technologies (3D ECM hydrogels and lung-on-a-chip-devices) to develop an advanced physiomimetic *in vitro* model of ARDS for the study of inflammatory processes and how they are related to MSCs therapies. Using such an advanced model, we have tested the individual contribution of cyclic stretch and lung ECM and the effect of lung-resident MSCs on the secretion of inflammation-related cytokines after bacterial lipopolysaccharide (LPS) challenge, as well as the impact of treatment with an anti-inflammatory drug (dexamethasone).

## 2 Materials and methods

Unless otherwise specified, all the reagents were obtained from Sigma Aldrich, Missouri, United States .

### 2.1 Decellularized extracellular matrix hydrogels

Lung hydrogels were developed from porcine lungs by following the protocol described in (Falcones et al., 2021). Five pig lungs were decellularized and the obtained powder was mixed to reduce batch-to-batch variability. Briefly, porcine lungs were decellularized by perfusion through the trachea and the vasculature with 0.1% Triton X-100% and 2% of sodium deoxycholate for 24 h each, and DNase and 1 M NaCl for 1 hour each. Decellularized lungs were afterward frozen at −80°C, freeze-dried (Telstar Lyoquest-55 Plus, Terrassa, Spain), and milled in liquid N_2_ (SPEX SamplePrep, New Jersey, United States) to obtain a fine powder. The resulting ECM powder was digested at a concentration of 20 mg/mL in HCl 0.01 M with porcine gastric mucosa pepsin at a 10:1 concentration at room temperature for 16 h. The digested solution was pH-adjusted to 7.4 ± 0.4 by using 0.1 M NaOH and incubated at 37°C for 20 min to produce the hydrogels. Final ECM concentration in the hydrogels ws ∼17 mg/mL.

### 2.2 Lung-on-a-chip devices fabrication

**FIGURE 1:**
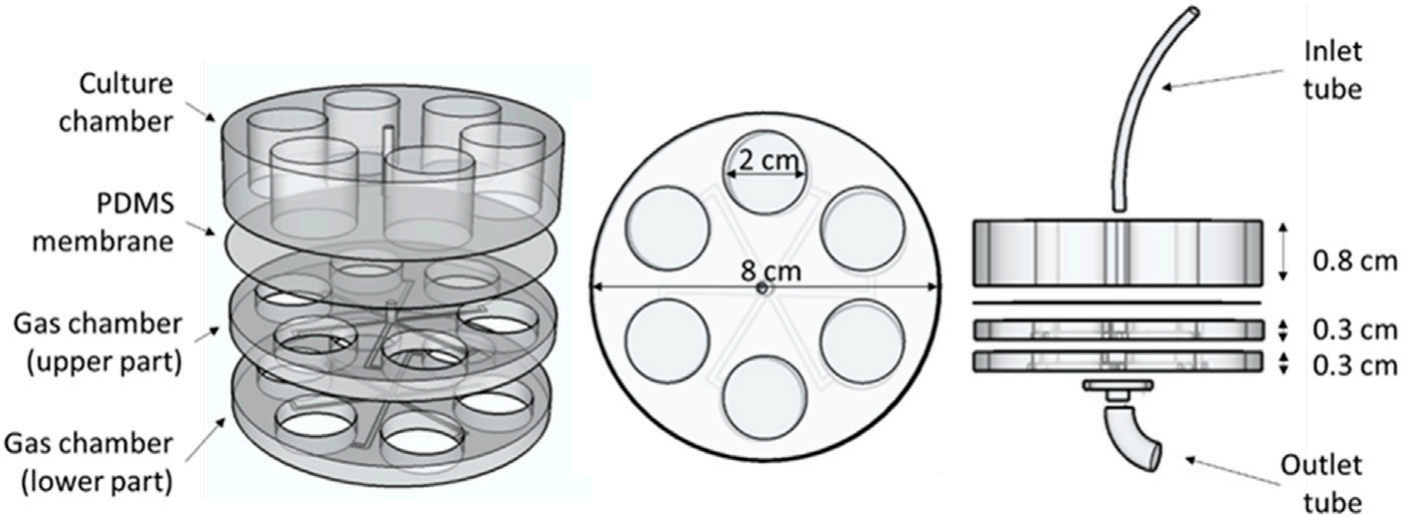
Chip design. Dimensions and different parts of the chip can be observed in this 3D representation. The upper part forming the culture chamber and the two lower parts forming the gas chamber can be easily distinguished, separated by the PDMS membrane.

The designed lung-on-a-chip devices were composed of three parts containing six holes, each one located concentrically to form the wells (Figure 1). A video with detailed instructions for fabricating a similar chip is open-source and available in the Supplementary Material of reference (Campillo et al., 2016). The upper part of the device, which was separated from the other two parts by a 380 µm thick gas permeable polydimethylsiloxane (PDMS) membrane (Gel-Pak, Hayward, CA, United States), was the culture chamber (cells and culture medium). The two other parts had channels in their lower part to allow for gas efflux (gas chamber). Gas entered the chip through a small tube inserted in a tiny central hole that perforates the culture chamber and the first part of the gas chamber. The air was distributed through the radial channels of the middle PDMS piece (upper part of the gas chamber) and diffused to the cells through the lower part of the membrane. Gas exited the chip through the radial channels of the lower PDMS piece (lower part of the gas chamber), coming back to the center of the chip, where gas found an exit through the perforated petri dish where the chip was located (Figure 1).

Once the gas had abandoned the chip, it was conducted through a tube to a proportional valve that opened and closed at 0.2 Hz frequency (mimicking the human physiological breathing rate). When the valve closed, the flexible PDMS membrane deflected until reaching a surface strain of up to 10%. The pressure inside the system was alleviated by a leakage that consisted of a small diameter tube (ID = 0.56 mm) (Cole Parmer, Illinois, United States) placed between the outlet of the chip and the proportional valve. The length of the leakage tube was individually adjusted for each fabricated chip to obtain the desired strain for a given pressure.

To fabricate the devices, negative molds of the parts were designed with the Ultimaker Cura software (Ultimaker, Utrecht, Netherlands) and printed with an Ultimaker S5 3D printer (Ultimaker, Utrecht, Netherlands) in polycarbonate material. PDMS prepolymer was mixed in a proportion 10:1 with the curing agent (Sylgard 184 kit, Dow Corning, MI) and poured into the previously printed molds. The resulting mixture was degassed in a bell jar vacuum desiccator (Kartell Labware, Noviglio, Italy) for 45 min and then placed in an oven (Selecta, Barcelona, Spain) for 2 h at 65°C. PDMS parts were carefully removed from the molds and the middle and the upper parts were perforated in the center with an awl for further introduction of the inlet tube. The middle and lower parts were bonded together concentrically after activating their surfaces with a hand-held corona (Electro Technic Products, Chicago, IL) at proximity (∼5 mm) for 1 minute at the highest voltage. As previously indicated, the PDMS membrane was also treated with the corona and attached to the already formed gas chamber. The upper part (culture chamber) was attached to the PDMS membrane surface using non-polymerized PDMS and then placed in the oven for 60 min at 65°C. In the meantime, a 60 cm^2^ petri dish (Techno Plastic Products AG, Trasadingen, Switzerland) was perforated in the center with a driller (1 cm diameter). The PDMS chip was then placed on the perforated petri dish and adhered to by using non-polymerized PDMS. A 1 mm (ID) inlet tube (Cole Parmer, Illinois, United States) was inserted in the central hole and sealed with non-polymerized PDMS. The whole assembled chip was placed in the oven for 60 min at 65°C. Lastly, a polycarbonate 3D-printed funnel-like piece was attached with glue to the lower part of the petri dish to connect the outlet tube.

The PDMS membranes of the devices fabricated for 3D cell culture were treated for proper adhesion of the hydrogels. Briefly, PDMS membranes were activated by introducing the chips in a plasma cleaner (PDC-002, Harrick Scientific Products Inc. Pleasantville, NY) for 2 min at maximum voltage and then introduced in the culture hood under the UV light for 10 min to sterilize the surface. APTES 10% was added for 1 h and 5 mM genipin (Challenge Bio Products Co., Taiwan) for 45 min. After each reagent, 3 PBS 1X washes of 5 min each were made. Finally, the chip was left to dry overnight.

### 2.3 Experimental setup and devices calibration and characterization

#### 2.3.1 Experimental setup

**FIGURE 2:**
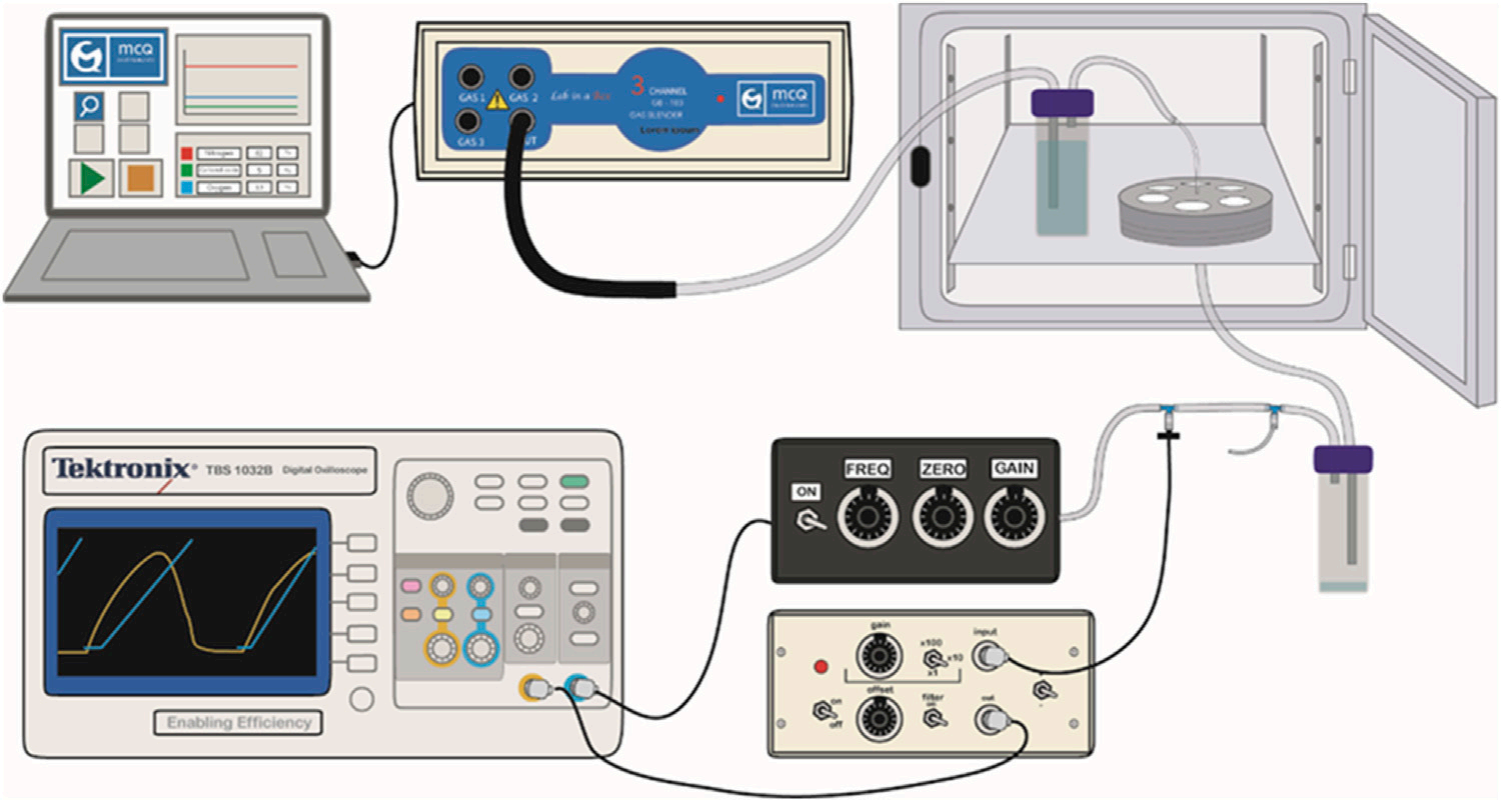
Experimental setup. The drawing shows how the air path from the gas blender, controlled by the software, to the valve, through the chip and the two water traps to initially humidify and finally de-humidify the gas. Cyclic stretch-generating pressure is measured along the experiment with the pressure sensor.

To support the specific gas mixture to the cultured cells, servo-controlled gas blenders (McQ, Virginia, United States) controlled by the Software Gas Mixture Creator (McQ, Virginia, United States) were employed. Previously humidified air went inside the chip through the inlet tube and then it was distributed through (Figure 2) the channels to reach the PDMS membranes where the cells were cultured. Then, the gas abandoned the chip through the outlet tube. After removing the humidity of the air, the conducting tube was connected to a valve for stretch amplitude and frequency control by using an incorporated pressure sensor (176PC14HD2, Honeywell, New Jersey, United States).

#### 2.3.2 Measurement of oxygen diffusion through 3D hydrogels

All the measurements were acquired inside a cell incubator at 100% humidity and 37°C. Before the measurements, chips were ventilated with 100% N_2_ for 30 min to displace the existing O_2_ in the hydrogel. An optical fiber oxygen sensor (Pyroscience, Aachen, Germany) was calibrated following the manufacturer’s instructions and attached to a specifically designed holder that allowed for micrometric-resolution vertical positioning. Measurements were performed with the sensor tip introduced 300 µm in the hydrogel. The gas mixture was changed to room air (20% O_2_ and 80% N_2_) to measure the oxygen diffusion time through the hydrogel. Measurements were repeated for different distances (100 µm steps) (Colom et al., 2014), and the diffusion time constant *τ* was calculated.

#### 2.3.3 Membrane and hydrogels strain calibration and characterization

The membrane deformation was calculated by modeling it as a spherical cup shape, where linear strain varies slightly across the membrane, but circumferential strain decreases parabolically to zero at the clamped edge (Campillo et al., 2016). The membrane experiences an equibiaxial linear strain (*ε*) that can be calculated as follows: the vertical deflection of the spherical cap (*h*) was calculated by the difference in height assessed with phase-contrast imaging using a confocal microscope with a motorized × 10 objective. At the same time, the radius (*r*) was known from chip design. The strain of the membrane was calibrated for different gas pressures by using Eq. 1:

$$\epsilon =\frac{2}{3}{\left(\frac{h}{r}\right)}^{2}$$ (1)

To assess that the stretch of the membrane was transmitted to the cells through the hydrogels, the latter were coated with 2 µm-diameter fluorescent carboxylated beads (Invitrogen, Oregon, United States). Epifluorescence images were taken at different pressures with a 10X objective. The displacement of the beads was computed by ImageJ as described in (Campillo et al., 2016).

### 2.4 Cells isolation and culture protocols

Primary lung mesenchymal stromal cells (LMSCs) and type 2 alveolar epithelial cells (ATIIs) were isolated from 180–250 g male Sprague-Dawley rats by following protocols described in (da Silva Meirelles, Chagastelles, and Nardi 2006) and (Guillamat-Prats et al., 2020) respectively, which were approved by the Ethical Committee for Animal Research of the University of Barcelona (Number 154/19, 02/10/2019).

For LMSCs extraction, rats were anesthetized with 1 g/kg urethane and euthanized by exsanguination through abdominal aorta excision. Lungs were perfused with 50 mL of PBS 1X through the right ventricle of the beating heart after cutting the left atrium. Lungs were excised *en bloc* with the heart. Lungs were sectioned into small pieces and digested in 10 mL of 250 U/mL collagenase (Gibco, Massachusetts, United States) solution prepared in DMEM with 10% HEPES for 1 h at 37°C under agitation. The resulting solution was filtered by a 250 µm mesh and then centrifuged at 400 g for 10 min (Rotina 380R, Hettich, Tuttlingen, Germany). The obtained pellet was resuspended in red blood cell lysis buffer (RBC) (BioLegend, San Diego, CA, United States) and was incubated at 4°C for 7 min. After that time, the reaction was stopped by adding PBS 1 × . Finally, cells were centrifuged at 350 g for 5 min and cultured in T-75 flasks for expansion. LMSCs up to passage seven were used for the experiments.

For ATIIs extraction, lungs were perfused with saline through the pulmonary artery and were resected *en bloc*. Five bronchioalveolar lavages were performed with 10 mL of PBS 1X to remove alveolar macrophages. Then, the lungs were digested with 50 mL of 0.25% trypsin through the airways for 30 min. Lungs were cut into small pieces, digested in a 100 units/mL DNase, and filtered through meshes of 100 µm and 40 µm of pore size. The filtered suspension was centrifuged through a percoll (GE HealthCare, Illinois, United States) gradient at 500 g for 20 min. The band containing the ATIIs was recovered and digested by DNase (20 units/mL). The solution was centrifuged for 15 min at 500 g. Then, the pellet was resuspended in DCCM-1 (Biological Industries, Kibbutz Beit Haemek, Israel) medium and cultured for 1 h. After that time, the medium containing ATII non-adherent cells was recovered and centrifuged for 10 min at 800 g. Cells were counted, seeded in the lung-on-a-chip devices and cultured in supplemented DCCM-1 medium (1% penicillin, streptomycin and amphotericin, 1% glutamine, and 10% FBS).

### 2.5 Acute respiratory distress syndrome-on-a-chip model

For the 3D culture, 3 × 10^5^ cells/mL of LMSC were resuspended in 500 µL of lung ECM pregel before gellification. Then, hydrogels were formed by placing the chips in the incubator for 20 min before adding 500 µl of supplemented DCCM-1. ATII cells were then cultured at a density of 10^6^ cells/well. All the experiments were performed at physiological oxygen levels (13%).

**FIGURE 3:**
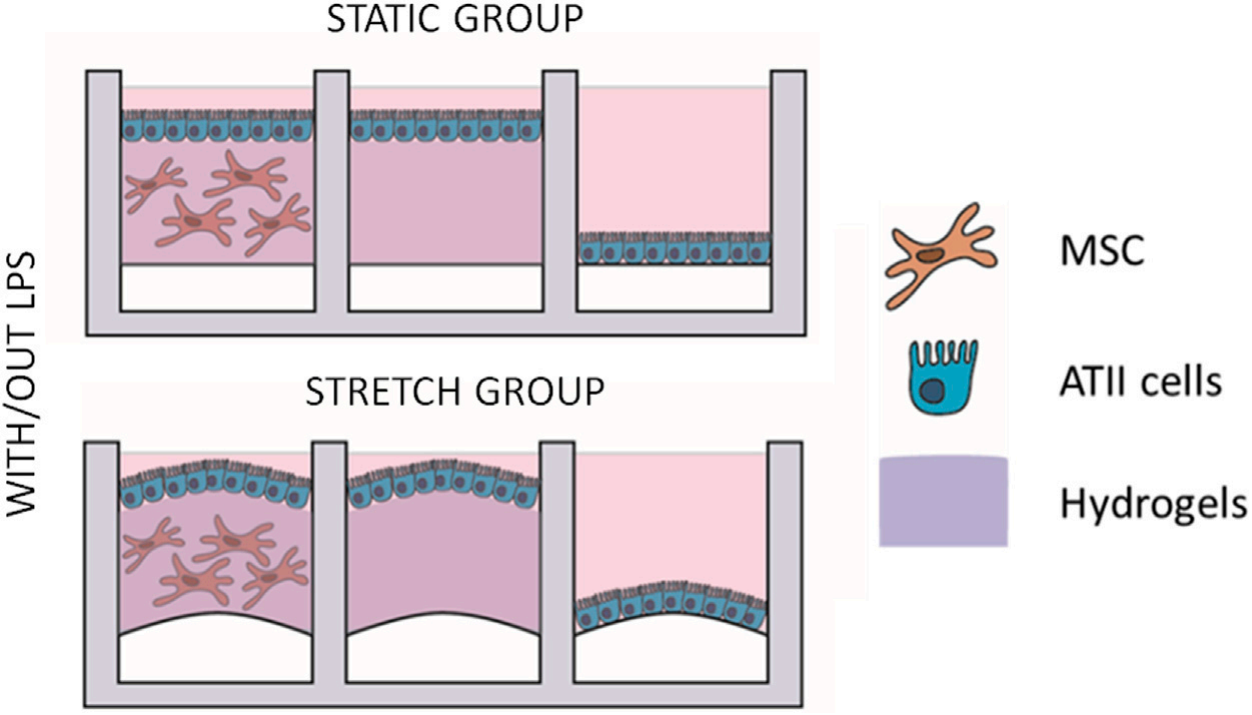
Four different experimental groups were designed. In every chip, three different cultures were performed: ATII-LMSC cocultured with/out 3D-cultured LMSC and an ATII monolayer on the hydrogels; ATII single culture monolayer on the hydrogel; and single culture ATII monolayer on the PDMS membrane. In every run of experiments, four chips were used, each of which was subjected to different conditions: static group, static group with an LPS hit, cyclic stretch group, and cyclic stretch group with an LPS hit.

A description of the experimental groups is shown in Figure 3: three groups were cultured in each device (co-culture of ATIIs and L-MSCs in 3D hydrogels, ATIIs cultured on top of hydrogels, and ATII cultured over the membrane). Half of the devices were subjected to cyclic stretch, and half of them to an LPS inflammatory hit (L2630 from *Escherichia Coli,* 1 µg/mL). Groups with co-cultures and cyclic stretch are referred to as “advanced physiomimetic model” (AM) while 2D cultures of ATIIs will be referred to as “traditional model” (TM). Cells were cultured under stretch for a total of 72 h (static conditions were maintained for the stretch controls groups), and the LPS hit was applied for 16 h. At the end of the experiment, the supernatants were collected for subsequent analysis. In the experiments involving the effect of an anti-inflammatory drug, 100 nM of dexamethasone were added for 32 h. Duplicates of six different samples were employed for these experiments (*n* = 6).

### 2.6 Multiplex ELISA analysis

ProcartaPlex Multiplex Immunoassays of the collected supernatants were performed according to the manufacturer’s instructions (ThermoFisher, Massachusetts, United States) for the following cytokines: macrophage inflammatory protein-2 (MIP-2), interleukins 1-beta, 6, and 10 (IL-1β, IL-6, IL-10), gamma interferon (INF-γ), alpha tumor necrosis factor (TNF-α), and vascular endothelial growth factor (VEGF). Data were acquired with Magpix (Luminex, Texas, United States) and processed using the ProcartaPlex Analysis App Software (ThermoFisher, Massachusetts, United States).

### 2.7 Immunohistochemistry

Cells were fixed with 4% paraformaldehyde for 30 min for the immunofluorescence experiments. Primary antibodies were incubated overnight, and secondary antibodies were incubated for 2 h at 37°C. Nuclei were stained with Hoechst 33,342 (ThermoFisher, Massachusetts, United States). To avoid unspecific binding, especially in the hydrogels, a blocking buffer consisting of 2% BSA (ThermoFisher, Massachusetts, United States) diluted in PBS 1X (Gibco, Massachusetts, United States) was employed for 40 min. After the primary antibody incubation, three washes of 5 min under orbital agitation were made. The primary antibodies used were rabbit anti-EpCAM and mouse anti-Vimentin; the secondary antibodies were goat anti-rabbit cy5 and goat anti-mouse Alexa Fluor 488. All antibodies were purchased from Abcam (Cambridge, United Kingdom). Images were taken with Nikon Confocal Eclipse Ti using a 20X Plan Fluor Multi-immersion objective (0.75 NA). Samples were excited at 488 nm and acquired at 515 nm for Vimentin images, and excited at 543.5 nm and acquired at 605 nm for EpCAM staining. Nuclear images were obtained at 450 nm when illuminating the sample at 408 nm.

### 2.8 Statistical analysis

Data are expressed as mean ± SE. Statistical analysis was performed with Prism software (GraphPad Software, California, United States). Differences in cytokine expression were studied using Student’s *t*-test, except in those cases where they did not follow a normal distribution, where a Mann-Whitney test was performed instead. The normal distribution of the samples was calculated by using a Kolmogorov-Smirnov test. Differences were considered significant for *p*-values < 0.05.

## 3 Results

### 3.1 Lung-on-a-chip device characterization

**FIGURE 4:**
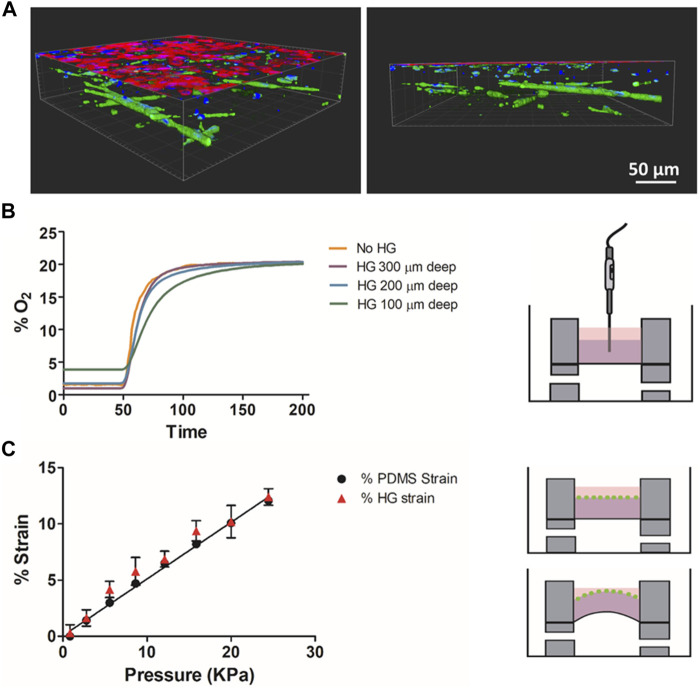
**(A)** Confocal images of the alveolar monolayer on top of the ECM hydrogels with LMSCs cultured inside. Alveolar cells (red) are stained for EpCAM and MSCs (green) are stained for vimentin **(B)** Oxygen diffusion measurements at different depths of the hydrogel and liquid medium (No HG). **(C)** Measured deformation of the membrane and the attached hydrogel at different pressures.

Three-dimensional images of the ATIIs monolayer on top of the ECM hydrogels with LMSCs cultured inside (Advanced model if stretched) are shown in Figure 4A. The efficient oxygen diffusion through the hydrogel when changing from 0% O_2_ to 20% O_2_ is shown in Figure 4B, where measurements were taken at different depths inside the hydrogel and compared with those acquired without hydrogel (culture medium) to study whether O_2_ was able to diffuse through the whole thickness. Measurements obtained with the fiber optic oxygen sensor showed that the time constant *τ* for the non-hydrogel measurement (just diffusion in the PDMS membrane) was ≈10 s, increasing by 40% for 300–200 µm depths and by 80% for the 100 µm depth. This indicates that the lung-derived hydrogels present a coefficient of diffusion for O_2_ that is quite similar to that of water, indicating these hydrogels are compatible with the three-dimensional culture and the precise control of oxygen partial pressure (Farré et al., 2018). Figure 4C shows the measured strain experienced by the PDMS membrane with the actual strain measured at the surface of the hydrogels calculated by the displacement of the attached fluorescent microbeads. It was observed that the strain applied to the flexible membrane was transmitted to the attached hydrogel 3D structures following a linear relationship with the applied pressure.

### 3.3 Response of the advanced physiomimetic model to the inflammatory hit

**FIGURE 5:**
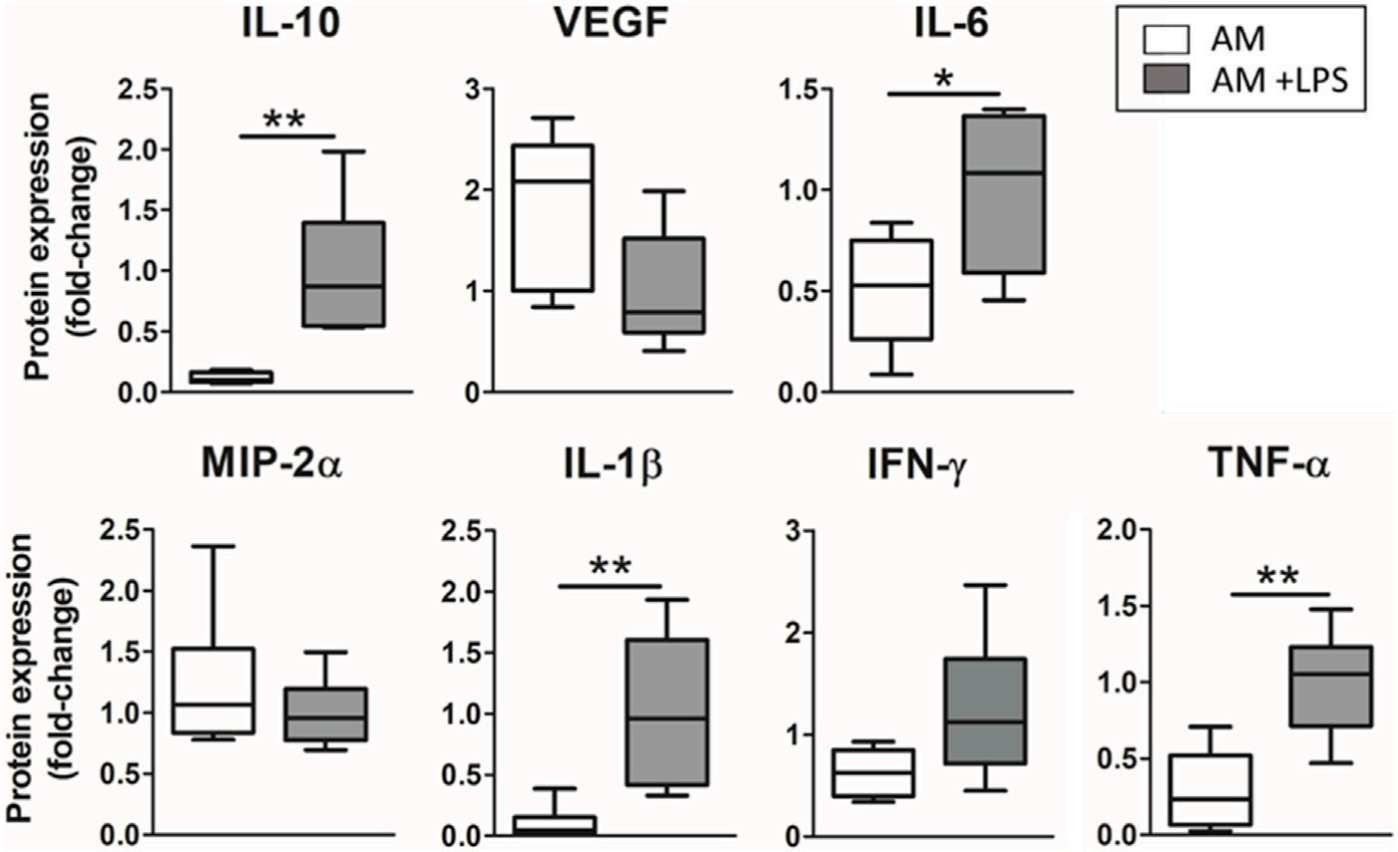
Response to LPS endotoxin of the advanced model (3D cultured LMSC in lung hydrogel with ATII cultured on top, subjected to cyclic stretch–Advanced Model).

The advanced model was subjected to an LPS endotoxin hit, and the release of inflammatory mediators was studied. LMSCs were cultured three-dimensionally while ATII were cultured on the top of the hydrogel, forming a monolayer, and cyclic stretch was applied. ELISA results on the secretion of cytokines for the advanced model developed when LPS endotoxin was added to the cultures are shown in Figure 5. The inflammatory hit produced a statistically significant increase in the secretion of inflammation-related cytokines IL-10 (8-fold; *p* = 0.003), IL-6 (2-fold; *p* = 0.02), IL-1β (10-fold; *p* = 0.0081) and TNF-α (3.5-fold; *p* = 0.0023) while no statistical difference was found in the secretion of VEGF, MIP-2α and IFN-γ.

**FIGURE 6:**
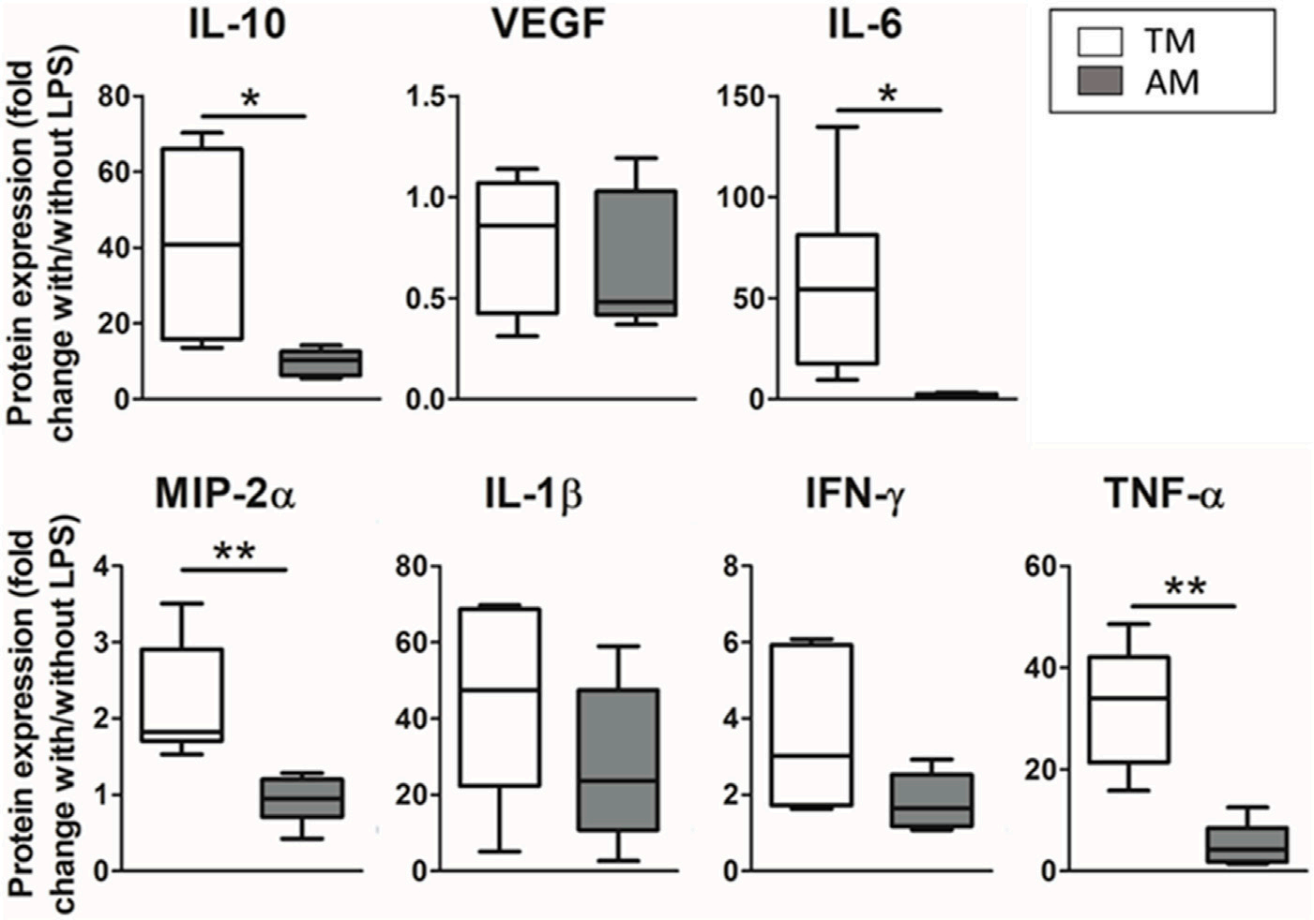
Different magnitude response of the advanced model (ATII cultured on hydrogels with 3D-cultured LMSC inside) compared to a traditional 2D culture model (ATII cell cultured on PDMS). Results were expressed as the ratio of cytokines expressed with/out the LPS hit, for either the traditional model or the advanced model.

Differences in the inflammatory response of the advanced model compared to the traditional 2D culture model are shown in Figure 6. Results are expressed as the ratio of the cytokines secreted with and without LPS within each group (TM or AM) to compare how both models are responsive. For all the measured cytokines, the response of the advanced model to the inflammatory hit was lower than in the traditional model; statistically significant differences were found for IL-10 (4-fold-fold; *p* = 0.015), IL-6 (29.5-fold-fold *p* = 0.024), MIP-2α (2.4-fold *p* = 0.0022), and TNF-α (6.5-fold-fold; *p* = 0.0014).

### 3.3 Effects of the 3D culture of mesenchymal stromal cells in hydrogels and cyclic stretch

**FIGURE 7:**
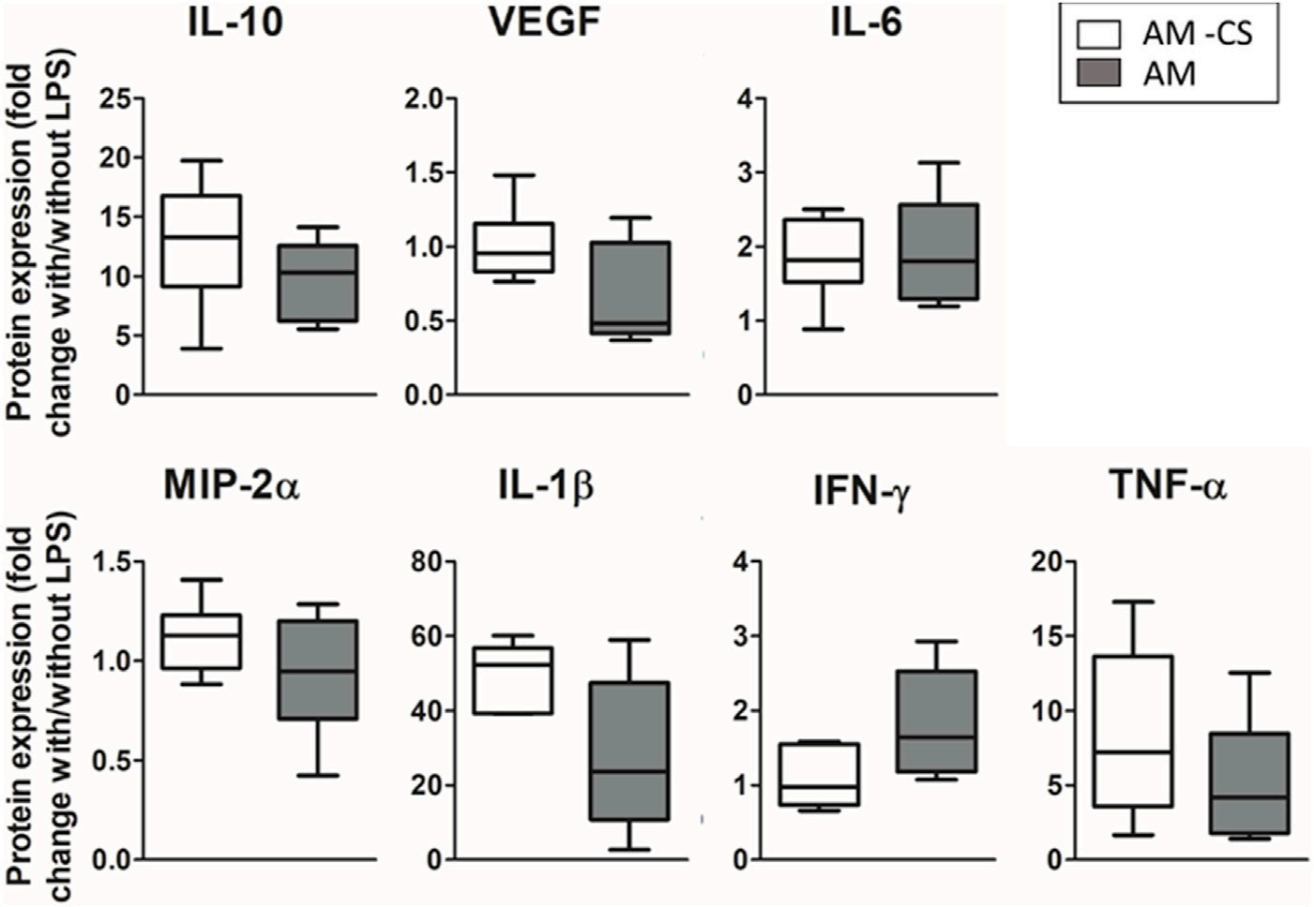
Effect of cyclic stretch. No statistical difference was observed in the secretion of inflammatory cytokines.

To study the effect of cyclic stretch alone in the physiomimetic model, four different groups were compared (with and without CS, each of which with and without LPS). Results in Figure 7 show the ratio of the amount of cytokines secreted with and without LPS either in the presence or absence of cyclic stretch. Cyclic stretch showed no impact on the response of the advanced model to the inflammatory hit (Figure 7). However, when studying the effect of CS in the absence of LPS, a statistically significant increase in the secretion of VEGF and a decrease in the secretion of IFN-γ (2-fold *p* = 0.046; 2-fold/0.55-fold, *p* = 0.02 respectively, data not shown) was observed when cyclic stretch was applied.

**FIGURE 8:**
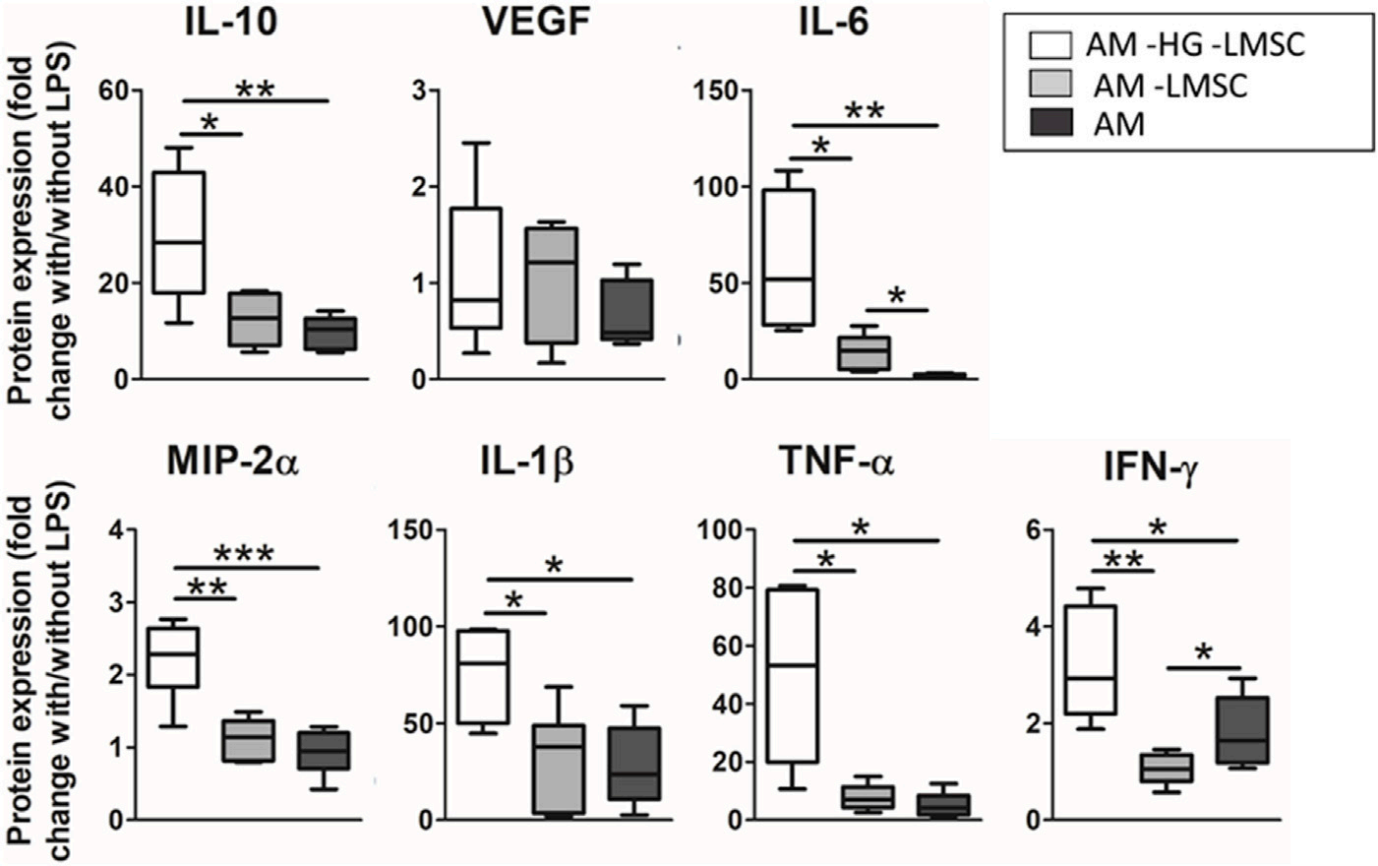
Effect of LMSC and hydrogel on the secretion of cytokines. Three groups were studied: ATII on PDMS with cyclic stretch (AM-HG-LMSC), ATII on hydrogel with cyclic stretch (AM-LMSC), and ATII on hydrogel with 3D LMSC cultured inside in the presence of cyclic stretch (AM).

The contribution of the lung hydrogel and the embedded LMSCs after application of the LPS hit was studied in three groups, as shown in Figure 8: 1) ATII on PDMS under cyclic stretch (AM–HG–LMSCs), 2) ATII on hydrogel under cyclic stretch (AM–LMSCs), and 3) ATII-LMSCs coculture in hydrogel subjected to cyclic stretch (AM). The effect of hydrogel significantly attenuated the secretion of all the cytokines but VEGF in response to the LPS endotoxin, showing inflammatory-suppressive properties. On the other hand, the presence of LMSCs significantly over-attenuated the secretion of IL-6 (7-fold; *p* = 0.0098) and promoted IFN-ɣ secretion (1.7-fold *p* = 0.032).

### 3.4 Response to dexamethasone

**FIGURE 9:**
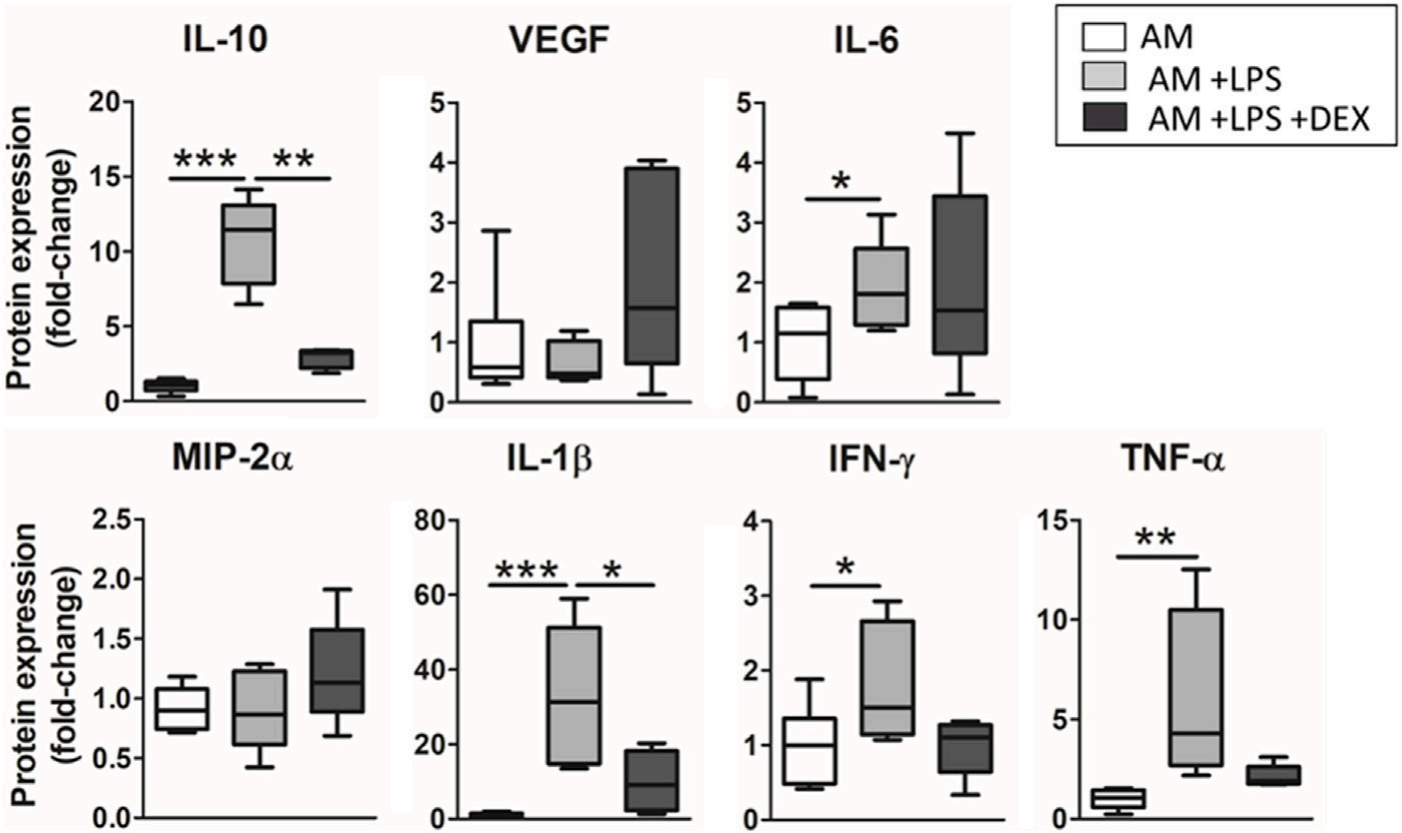
Anti-inflammatory effects of dexamethasone were studied in the advanced model. There was observed a general trend in recovering the expression levels observed before the inflammatory hit, which were statistically significant in the case of IL-10 and IL-1β.

Results on the secretion of cytokines when dexamethasone was added to the advanced model are shown in Figure 9. The treatment induced a trend to recover the levels of cytokine expression observed before the inflammatory hit in TNF-α and IFN-ɣ, and a significant decrease in the secretion of IL-10 (3.7-fold decrease, *p* = 0.0014) and IL-1β (3.2-fold decrease *p* = 0.0407).

## 4 Discussion

Experimental *in vitro* models of severe respiratory diseases such as ARDS are important to better understand the basic pathophysiologic mechanisms involved and the potential effects of drug treatments. However, the conventional model based on cells cultured on a plate is an extreme simplification of the microenvironment experienced by cells *in vivo* and it has shown several limitations when trying to mimic *in vivo* results. Therefore, there is an urgent need for improved models that are more realistic in reproducing the cells’ physical, chemical, and biological conditions in the lung. The work presented herein shows the feasibility of developing a physiomimetic model for *in vitro* ARDS studies by combining organ-on-a-chip technologies and ECM-derived hydrogels for 3D cell culture. Specifically, the response of the developed model to an endotoxin-mediated inflammatory hit showed marked differences compared to the traditional plastic-based 2D models, the latter apparently overestimating the cytokine response. In this context, the advanced model (3D architecture with biophysical stimuli) described herein is a novel contribution to setting more physiomimetic approaches for studying the mechanisms of ARDS and potential treatments.

The developed model overcomes previous limitations of *in vitro* studies on ARDS by recreating a physiomimetic environment and by using epithelial and stromal cells in co-culture. Device molds were fast prototyped by 3D printing; hence the dimensions of the devices can be easily modified depending on the requirements of each study. The developed chips were compatible with optical microscopy, which eased the calibration of the membrane stretch as a function of the applied pressure. This aspect opens the door for future studies incorporating real-time imaging of the cultured cells in the experiments. We incorporated 3D cultures into the lung-on-a-chip devices developed by attaching cell-laden ECM hydrogels to the PDMS stretchable membrane previously treated with APTES and genipin. No detachment of the hydrogels from the membranes was observed during the experiments. Still, there was an open question for lung-derived ECM hydrogels for organ-on-a-chip devices regarding oxygen diffusivity and stretch transmission from the device to the 3D scaffold (Otero et al., 2021). Oxygen was expected to diffuse quickly in the hydrogels as they are composed mainly of water, and results obtained in the experiments conducted here confirmed that hypothesis. The diffusion of oxygen has been shown to highly impact cell response (Marhuenda et al., 2019); thus, results obtained in our experiments demonstrate the suitability of their use for three-dimensional culturing. Regarding the transmission of the stretch from the membrane to the hydrogel, we observed that it was correctly experienced by the 3D hydrogel by measuring distances between beads, a method adapted from 2D cultures stretch calibration (Trepat et al., 2004). Nevertheless, it should be noted that changing the geometries and the mechanical properties of the hydrogels could modify this stretch transmission, especially if the hydrogels were softer (as they usually are when using lower ECM powder concentrations in their preparation) (Pouliot et al., 2016; de Hilster et al., 2020; Martinez-Garcia et al., 2021). In our case, the results showed that in the physiomimetic model, the 3D co-cultures were oxygenated and stretched similarly as in traditional 2D models. In this way, the analysis of the contribution of the 3D microarchitecture can be more robustly analyzed from the conducted experiments (Nonaka et al., 2016).

The present study was carried out with freshly isolated rat alveolar cells and primary rat lung mesenchymal stromal cells. The use of primary cells is highly advantageous compared to previous works where cancer cell lines or immortalized ATIIs that have lost some of their typical characteristics were employed (Chen et al., 2019; Abate et al., 2010; Willis et al., 2005). The possibility of using primary ATIIs was eased by the employment of lung-derived hydrogels, which are more suitable substrates for cell culture than culture plates, allowing alveolar cells not only to form monolayers but also to maintain the secretion of surfactant B and C proteins for longer times (Esther Marhuenda et al., 2022), which is of high importance when studying ARDS (Wang S et al., 2021). The results obtained using primary alveolar cells are expected to be more easily translational than those using A549 cells (Chen et al., 2019), which are usually used as a surrogate of ATIIs in *in vitro* studies. Also, all the experiments shown herein were performed at physiological oxygen concentrations (13%) (Wild et al., 2005; Brahimi-Horn and Pouysségur 2007), avoiding that cells were subjected to the typical hyperoxic conditions in traditional culture settings (≈20% oxygen), which can induce the secretion of reactive oxygen species (Stuart et al., 2018) thereby potentially altering the inflammatory response to LPS challenge and to drug treatment. For the sake of experimental simplicity and similarly as most published *in vitro* research on ARDS, this study aimed at designing and testing an advanced physiomimetic model has been carried out using animal samples (rat cells and porcine ECM). However, the model can be exactly reproduced by employing cells and ECM from human lungs, thereby avoiding any effect owing to interspecies differences (Mercer and Crapo 1990; Mercer, Russell, and Crapo 1994).

Results on the inflammatory response of the developed model to a classic inflammatory hit (i.e., LPS) modeling ARDS were in line with what has been observed in previous *in vitro* and *in vivo* studies (Huh et al., 2018; Voiriot et al., 2017; Li et al., 2020; Cabrera-Benítez et al., 2016; Peñuelas et al., 2013). As expected, the inflammatory response observed in the advanced model was attenuated, indicating that the presence of LMSCs and the incorporation of different biophysical stimuli play a protective role in response to an endotoxin hit. The overresponse observed in traditional models can then be a result of culturing cells on petri dishes, which have very different physical properties and biochemical environment than the model developed herein. Interestingly, the fact that our model is less sensitive to the LPS hit could ease the development of future studies with different endotoxin doses aiming to model different degrees of disease severity.

By separately studying the contribution of the different stimuli, very interesting data were obtained regarding the decreased responsiveness to the inflammatory challenge. No significant differences were observed in the secretion of cytokines by the effect of cyclic stretch alone. This could be because the impact of cyclic stretch in a physiomimetic model is moderated and shielded by the other factors present in the advanced model. The anti-inflammatory effect of cyclic stretch has been previously reported (Fang et al., 2018) in a much more responsive model, which was also observed in our control experiments in 2D, and also reported to impact on cell fate in alveolar and mesenchymal cells (Heise et al., 2011). On the other hand, culturing cells in lung ECM-derived hydrogels was the factor of major importance in attenuating the inflammatory response in the developed model. Culturing cells using a substrate with lung parenchyma-like stiffness (Falcones et al., 2021) and a more physiomimetic and complex biochemical composition seem to offer primary alveolar cells and lung mesenchymal stromal cells a protective environment that attenuated the inflammatory response. It should be noted that which components of the ECM present in the hydrogels are playing a major role in this alteration of the inflammatory response is still an open question. Indeed, depending on the ECM decellularization and digestion (Pouliot et al., 2020) protocols, the final composition of the hydrogels may vary. Recent mass spectrometry studies on porcine-derived ECM hydrogels composition (Kim et al., 2022) showed a high number of ECM proteins present in the hydrogels but a high variability between individuals. However, it has been shown that the decellularization protocol presented herein preserves at least partially elastin, collagen and GAGs (Pouliot et al., 2016), and that type I collagen in the lung is mostly preserved by the digestion time used (Pouliot et al., 2020). Finally, the LMSCs played an important role in the inflammatory context due to the cytokine secretion and the interaction with the epithelial monolayer. As previously reported (Chen et al., 2019), the presence of LMSCs in the model altered the inflammatory response, but, interestingly, the results showed that the effect of lung hydrogel itself has a much greater impact than the presence of LMSCs.

The main limitation in correlating the cytokine expression from results obtained *in vitro* and *in vivo*, is the fact that most *in vitro* cultures are performed under quite unrealistic conditions (attending to the complex physiological biochemical and biophysical environment) and more importantly, to the interactions of different cell types (Li et al., 2020; Cabrera-Benítez et al., 2016). The expression of IL-10, IL-8 (its murine counterpart is MIP-2α), and IL-6 have a high clinical significance in ARDS patients, as high values of these cytokines are clearly related to the severity of the disease. IL-6 has particular interest due to the link between its increase and a fatal prognosis, being related to increased lung compliance, the altered levels of Pa $o_{2}$ /Fi $o_{2}$ , and the need for mechanical ventilation (Stukas et al., 2020; Wang J et al., 2021). Nowadays, the lack of a gold-standard treatment for ARDS patients is a matter of concern. Glucocorticoids such as dexamethasone are often used to improve ARDS patients’ outcomes, but there is still controversy about its benefits in all patients, mainly owing to the heterogeneity in the population receiving the treatment. For example, only severe cases of disease caused by COVID benefited from short-term low-dose treatment (Sterne et al., 2020; van Paassen et al., 2020). In addition, it is broadly accepted that MSCs present immunomodulatory properties, and as such, they have been proposed as a therapy for ARDS (Guillamat-Prats et al., 2020; Liu et al., 2020). However, little is known about how the presence of dexamethasone can modify these immunomodulatory properties. Results presented herein, although limited, suggest that the effect of drugs such as dexamethasone should be better studied *in vitro* by using physiomimetic models like the ones developed in the present work. Studies with dexamethasone-treated epithelial cells in much more responsive 2D models showed a decrease in the proinflammatory cytokines (Chen et al., 2021; Patil et al., 2018), but the results presented in this work are the first ones conducted in a 3D model with more physiological responsiveness to the LPS hit. Regarding MSCs, results are more controversial: while some *in vitro* studies have shown a decrease in cytokines and chemokines secreted by cytokine-stimulated LMSCs under dexamethasone effects (Wallace et al., 2001; Kim, Cheng, and Kim 1999), *in vivo* studies point that dexamethasone could be abrogating the anti-inflammatory effect of MSC (Chen et al., 2014; Wang et al., 2018). This impairment of the LMSC anti-inflammatory properties by dexamethasone could explain why a drastic decrease in the proinflammatory cytokines was not observed in the developed model: while dexamethasone was decreasing the secretion of proinflammatory cytokines by epithelial cells, it could be impairing the anti-inflammatory properties of LMSCs. Therefore, the levels of cytokines measured in the advanced model may be the result of a balance, showing a scenario much more similar to what is occurring *in vivo*, which was the major aim of the developed physiomimetic model for ARDS. The results obtained here were more aligned with some *in vivo* studies performed in rats, where LPS showed an increase in cytokine expression while the treatment with dexamethasone decreased these levels but not completely recover the levels before the LPS hit (Qin and Qiu 2019; Li, Whiteman, and Moore 2009).

In conclusion, this work suggests that the developed model of ARDS-on-a-chip responds to an LPS challenge and partially recovers the secretion of cytokines after anti-inflammatory drug treatment. Thus, this novel model opens the door for further *in vitro* research on developing different therapeutic strategies for ARDS treatment. Although it is impossible that any *in vitro* model fully mimics the inflammatory process occurring in the lungs during ARDS, the advanced model describes a step forward. Lung-on-a-chip devices based on PDMS membranes aimed at analyzing the effects of physical parameters such as stretch on cells have been developed during the last decade. The setting presented herein corresponds to what is known as second generation of lung-on-a-chips (Zamprogno et al., 2021). The main concept is to culture the cells in a physiological matrix instead on functionalized PDMS. In the present work, we did not use a combination of ECM proteins but a biomaterial exclusively made from native ECM components, being therefore a step forward the previous settings. Specifically, our approach allows for studying how alveolar epithelial cells respond to an inflammatory stimulus and how the resident lung mesenchymal stromal cells can play a role in it when cells are in their native ECM and subjected to realistic cyclic stretch. Moreover, it is a versatile model facilitating that different cell types could be included to further study crosstalk mechanisms among the other players involved in the inflammatory process of ARDS. Interestingly, the possibilities in tuning the model makes it suitable for expanding its use to study in detail respiratory diseases other than ARDS, including applications in high-throughput drug testing for new treatment developments.

## Data Availability

Data supporting the findings of this study are available from the corresponding author upon reasonable request.
